# Dispensable roles of Gsdmd and Ripk3 in sustaining IL-1β production and chronic inflammation in Th17-mediated autoimmune arthritis

**DOI:** 10.1038/s41598-021-98145-y

**Published:** 2021-09-21

**Authors:** Yusuke Takeuchi, Daiya Ohara, Hitomi Watanabe, Noriko Sakaguchi, Shimon Sakaguchi, Gen Kondoh, Akio Morinobu, Tsuneyo Mimori, Keiji Hirota

**Affiliations:** 1 Laboratory of Integrative Biological Science, Institute for Frontier Life and Medical Sciences, Kyoto University, 53 Kawahara-cho, Shogoin, Sakyo-ku, Kyoto, 606-8507 Japan; 2 Department of Experimental Immunology, Immunology Frontier Research Center, Osaka University, Osaka, 565-0871 Japan; 3 Department of Rheumatology and Clinical Immunology, Graduate School of Medicine, Kyoto University, Kyoto, 606-8507 Japan; 4 Ijinkai Takeda General Hospital, Kyoto, 601-1495 Japan

**Keywords:** Autoimmunity, Cell death and immune response, Inflammation

## Abstract

Programmed necrosis, such as necroptosis and pyroptosis, is a highly pro-inflammatory cellular event that is associated with chronic inflammation. Although there are various triggers of pyroptosis and necroptosis in autoimmune tissue inflammation and subsequent lytic forms of cell death release abundant inflammatory mediators, including damage-associated molecular patterns and IL-1β, capable of amplifying autoimmune Th17 effector functions, it remains largely unclear whether the programs play a crucial role in the pathogenesis of autoimmune arthritis. We herein report that Gasdermin D (Gsdmd) and receptor interacting serine/threonine kinase 3 (Ripk3)—key molecules of pyroptosis and necroptosis, respectively—are upregulated in inflamed synovial tissues, but dispensable for IL-1β production and the development of IL-17-producing T helper (Th17) cell-mediated autoimmune arthritis in SKG mice. *Gsdmd*^−/−^, *Ripk3*^−/−^, or *Gsdmd*^−/−^ *Ripk3*^−/−^ SKG mice showed severe arthritis with expansion of arthritogenic Th17 cells in the draining LNs and inflamed joints, which was comparable to that in wild-type SKG mice. Despite the marked reduction of IL-1β secretion from *Gsdmd*^−/−^ or *Ripk3*^−/−^ bone marrow-derived DCs by canonical stimuli, IL-1β levels in the inflamed synovium were not affected in the absence of Gsdmd or Ripk3. Our results revealed that T cell-mediated autoimmune arthritis proceeds independently of the pyroptosis and necroptosis pathways.

## Introduction

Programmed necrosis associated with tissue inflammation is a newly reported concept of a lytic form of cell death that has emerged over the past few decades^1^. Pyroptosis and necroptosis, two distinct forms of programmed necrosis, not only play a crucial role in the host defense against infectious pathogens^1–3^, but have also been highlighted in various pathological conditions^2,4^. Upon TNF signaling, inflammasome activation and cellular stress responses, key pyroptotic and necroptotic pathways, respectively, are initiated and finally result in the abundant release of pro-inflammatory components, including IL-1, IL-18, and damage-associated molecular patterns (DAMPs), which can provoke and augment immune reactions and tissue inflammation^1^. Gasdermin D (Gsdmd) has recently been identified as the executioner of pyroptosis and the cleavage of Gsdmd in its linker domain mediated by inflammasome and specific caspase activation induces its oligomerization and forms pores in the cell plasma membrane, leading to lytic cell death^5,6^. On the other hand, receptor-interacting serine/threonine kinase 3 (Ripk3) is a specific key molecule of necroptosis to activate the pseudokinase mixed lineage kinase domain-like (MLKL) protein, which finally executes cell death^7,8^. Recent reports using *Gsdmd*^−/−^ or *Ripk3*^−/−^ mice revealed a pivotal role of pyroptosis and necroptosis in several tissue injuries and disease models, such as non-alcoholic hepatitis, familial Mediterranean fever (FMF), experimental autoimmune encephalomyelitis (EAE), cerulean-induced pancreatitis, ischemia/reperfusion injury, and kidney transplantation^9–14^. Thus, the aberrant activation of programmed cell death exacerbates the tissue inflammation associated with acute injuries and innate cell activation. However, it remains elusive whether programed necrosis plays a crucial role in the pathogenesis of T cell-mediated autoimmune diseases such as rheumatoid arthritis (RA).

RA, one of the most common human autoimmune diseases, affects approximately 1% of the world population, and is characterized by bone-destructive chronic polyarthritis^15^. Although the precise pathophysiology of RA remains to be determined, it is widely appreciated that CD4^+^ T helper (Th) cells play a pivotal role in the pathogenesis of RA; this is supported by accumulating evidence, for example, MHC class II haplotype human leukocyte antigen (HLA)-DRB1 was identified as the strongest disease susceptibility gene and a high efficacy of cytotoxic T lymphocyte antigen 4 (CTLA4)-Ig blocking T cell co-stimulatory molecules for the treatment of RA^15–17^. Chronic synovial inflammation can accompany the cell death of proliferated fibroblast-like synoviocytes (FLSs) and recruited immune cells as well as pro-inflammatory cytokine production and the release of DAMPs, presumably by pyroptosis and necroptosis due to various triggers that are present in inflamed joints, including TNF and endogenous inflammasome ligands. Although it is considered that these events can amplify chronic inflammation and joint pathology^18^, it is unclear how a type of programed necrosis contributes to autoimmune arthritis.

SKG mice, a murine model of RA, spontaneously develop IL-17-producing T helper (Th17) cell-dependent autoimmune arthritis, resembling the immunopathology of chronic synovial inflammation in RA patients^19,20^. We have previously shown that DAMPs, including the alarmin IL-33, augment chronic inflammation in the inflamed joints of SKG mice, at least in part by stimulating tissue-resident synovial innate lymphoid cells (ILCs)^21^. Effector Th17 cells together with activated FLSs and ILCs orchestrate chronic arthritis by secreting abundant proinflammatory cytokines, including IL-6, IL-17, GM-CSF, and TNF, and these inflammatory cellular responses and their signaling pathways potentially trigger necrosis. Because lytic forms of programed necrosis can release abundant inflammatory mediators, including DAMPs and IL-1β, which is capable of amplifying the autoimmune Th17 effector functions and joint inflammation, in this study, SKG mice were used as a suitable model to investigate whether programed necrosis contributed to the differentiation and expansion of arthritogenic Th17 cells and the development of autoimmune arthritis.

## Results

### The generation of *Gsdmd*^−/−^ SKG mice and CD4^+^ T cell profiles in Peyer’s patches under a homeostatic state

**Figure 1:**
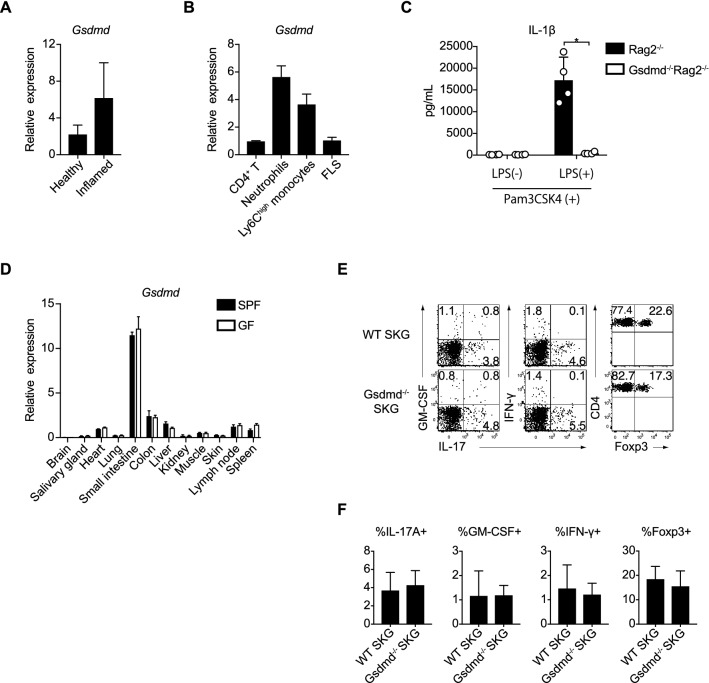
The *Gsdmd* expression in the tissues and inflammatory cells and CD4 + T cell profiles in *Gsdmd*^*-/-*^ SKG mice. (**A**) Quantitative RT-PCR to analyze the expression of *Gsdmd* in whole synovial tissues of healthy and inflamed joints (n = 4 each). (**B**) Quantitative RT-PCR to detect the *Gsdmd* mRNA expression in CD4^+^ T cells, CD11b^+^Ly6G^+^ neutrophils, CD11b^+^Ly6G^-^Ly6C^high^ inflammatory monocytes and CD45^-^CD31^-^ Podoplanin^+^ FLS in inflamed joints (n = 4 each). (**C**) The IL-1β concentration of culture supernatant of Pam3CSK4-primed BMDCs at 16 h after LPS transfection (n = 4 each). (**D**) Quantitative RT-PCR to detect the expression of *Gsdmd* in the indicated organs of BALB/c mice housed under SPF or germ-free conditions (n = 4 each). (**E**,**F**) Flow cytometry of IL-17A^+^, GM-CSF^+^, IFN-γ^+^ and Foxp3^+^ cells in CD4^+^ T cells in Peyer’s patches of age-matched healthy WT and *Gsdmd*^−/−^ SKG mice (n = 8 each). The vertical bars denote the SD in panel **(A–C,F)** and SEM in panel **(D)**. *P < 0.05. Data are pooled from two independent experiments in panels (**A–D)** and three independent experiments in panel **(F)**.

To investigate the role of Gsdmd in the pathogenesis of autoimmune arthritis, we first evaluated the *Gsdmd* expression in whole synovial tissues from healthy and arthritic SKG mice. When arthritis was induced in SKG mice, the expression of *Gsdmd* tended to be upregulated in the inflamed joints (Fig. 1A). We next assessed the *Gsdmd* expression in CD4^+^ T cells, CD11b^+^Ly6G^+^ neutrophils, CD11b^+^Ly6G^-^Ly6C^high^ inflammatory monocytes and CD45^-^CD31^-^Podoplanin^+^ FLSs sorted from the inflamed joints, each of which are the key cellular components that can augment chronic joint inflammation. The expression of *Gsdmd* in neutrophils and monocytes was higher in comparison to CD4^+^ T cells and FLSs, indicating the possible relevance of Gsdmd in these myeloid cells to the pathophysiology of SKG arthritis (Fig. 1B). We then generated *Gsdmd*^−/−^ SKG mice on a BALB/c background by using CRISPR/Cas9 system to examine the effects of Gsdmd on the differentiation and maintenance of CD4^+^ T cell subsets in homeostatic and inflammatory conditions (Supplementary Fig. 1A). As reported previously in a C57BL/6 background^5,10,11^, *Gsdmd*^−/−^ SKG mice grew up normally like wild-type (WT) SKG mice and the deletion of Gsdmd had no effect on their development, lifespan, or fertility (data not shown). Loss of the Gsdmd function in pyroptosis was confirmed by in vitro assay of LPS transfection into bone marrow-derived dendritic cells (BMDCs) from *Gsdmd*^−/−^ *Rag2*^−/−^ mice, which showed a remarkable reduction of the capacity for IL-1β release in comparison to BMDCs from WT *Rag2*^−/−^ mice (Fig. 1C), as previously reported using other *Gsdmd*^−/−^ strains^5^. To explore whether the physiological Gsdmd expression levels have any impact on CD4^+^ T cell profiles under a homeostatic condition, we assessed the *Gsdmd* expression in normal tissues from BALB/c mice maintained under specific-pathogen-free (SPF) conditions and found that the small intestine is an organ that constitutively expresses the high levels of *Gsdmd*, and the expression was found to be independent of the gut microbiota and bacterial components based on the comparable expression of *Gsdmd* in germ-free (GF) mice (Fig. 1D). Because various CD4^+^ T cell subsets reside in Peyer’s patches of the small intestine, we examined the proportions of physiological Th1, Th17 and regulatory T (Treg) cells in Peyer’s patches of WT and *Gsdmd*^−/−^ SKG mice under SPF conditions. *Gsdmd*^−/−^ SKG mice showed almost the same CD4^+^ T cell profiles in terms of the expression of IL-17, GM-CSF, IFN-γ and Foxp3 in comparison to WT SKG mice (Fig. 1E, F). Taken together, these results indicated that Gsdmd had little impact on the differentiation and maintenance of physiological CD4^+^ T cell subsets under a homeostatic state.

### Gsdmd is dispensable for the induction and development of SKG arthritis

**Figure 2:**
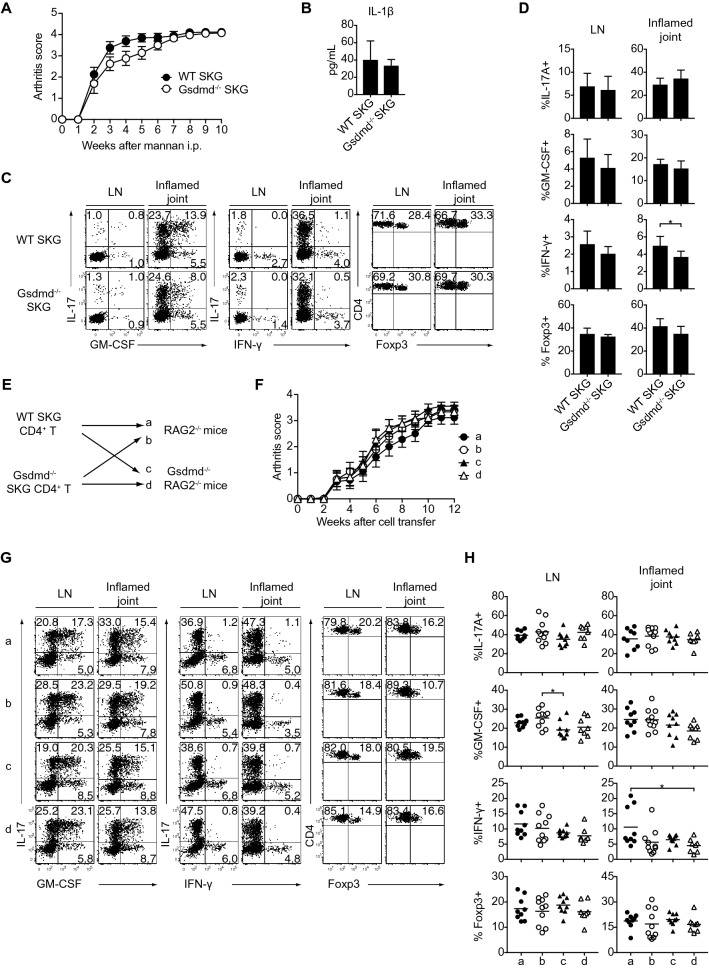
The dispensable role of Gsdmd in the development of autoimmune arthritis. (**A**) The arthritis scores of WT and *Gsdmd*^−/−^ SKG mice (n = 8 each) after the injection of mannan (20 mg). (**B**) The IL-1β concentration of synovial tissues from inflamed joints. **(C,D)** Flow cytometry of IL-17A^+^, GM-CSF^+^, IFN-γ^+^ and Foxp3^+^ cells in CD4^+^ T cells in draining LNs and inflamed joints of WT and *Gsdmd*^−/−^ SKG mice at 10 weeks after mannan injection (n = 8 each). (**E**) Experimental design of adoptive transfer of CD4^+^ T cells from WT or *Gsdmd*^−/−^ SKG mice into *Rag2*^−/−^ or *Gsdmd*^−/−^ *Rag2*^−/−^ mice. (**F**) The arthritis scores of the four groups (a-d) shown in (**E**) (n = 9–10 each). (**G**,**H**) Flow cytometry of IL-17A^+^, GM-CSF^+^, IFN-γ^+^ and Foxp3^+^ cells in CD4^+^ T cells in draining LNs and inflamed joints of the four groups of mice at 12 weeks after cell transfer (n = 8–10 each). The vertical bars denote the SD in panels (**B**,**D**) and SEM in (**A**,**F**). The horizontal line in panel (**H)** denotes the mean. *P < 0.05. Data are pooled from two independent experiments in panels **(A,B,D,F,H)**.

We induced arthritis in WT and *Gsdmd*^−/−^ SKG mice and assessed the clinical phenotypes and the development of arthritogenic Th17 cells. The severity of arthritis did not differ between the two groups of mice (Fig. 2A). Notably, the levels of IL-1β in the synovium from the inflamed joints of the two groups were comparable, suggesting that IL-1β could be produced in a Gsdmd-independent mechanism (Fig. 2B). We then assessed the effects of Gsdmd on CD4^+^ T cell profiles after the induction of arthritis. The frequencies of IFN-γ-, GM-CSF-, IL-17-, and Foxp3-expressing subsets in CD4^+^ T cells from inflamed joints or draining lymph nodes (LNs) were almost unchanged between the groups (Fig. 2C, D). We also evaluated the onset and severity of arthritis induced by the adoptive transfer of CD4^+^ T cells from WT or *Gsdmd*^−/−^SKG mice into *Rag2*^−/−^ or *Gsdmd*^−/−^*Rag2*^−/−^ mice, which enables us to dissect the specific role of Gsdmd in CD4^+^ T cells and non-CD4^+^ T cells in recipient mice, separately (Fig. 2E). Like the arthritis phenotype in *Gsdmd*^−/−^ SKG mice, the disease progression curves of arthritis were comparable in the four groups. Furthermore, the differentiation and expansion of arthritogenic Th17 cells in inflamed joints and draining LNs were not affected in Gsdmd-deficient conditions (Fig. 2F–H). These data demonstrated that Gsdmd is dispensable in the induction of arthritogenic Th17 cells and the development of chronic arthritis in SKG mice.

### The generation of *Ripk3*^−/−^ SKG mice and CD4^+^ T cell profiles in Peyer’s patches under a homeostatic state

**Figure 3:**
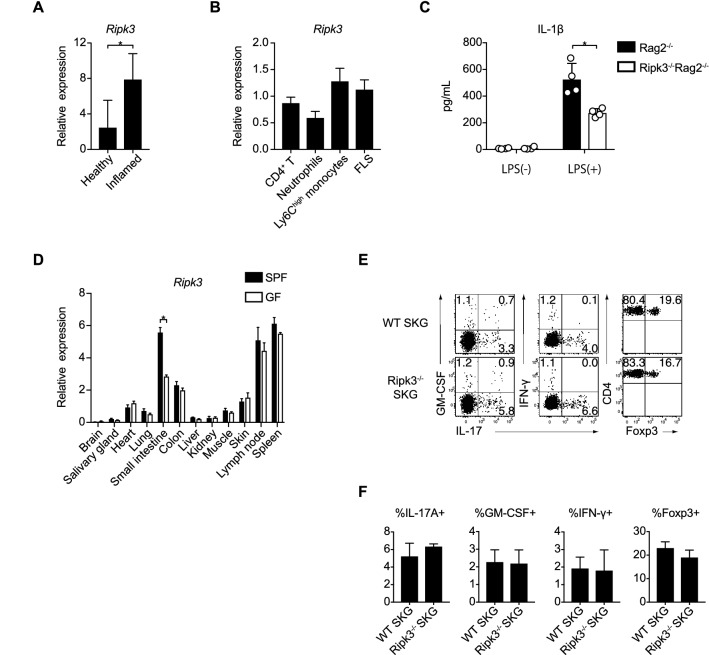
The *Ripk3* expression in tissues and inflammatory cells and CD4 + T cell profiles in *Ripk3*^−/−^ SKG mice. (**A**) Quantitative RT-PCR to detect the expression of *Ripk3* in whole synovial tissues of healthy and inflamed joints (n = 4 each). (**B**) Quantitative RT-PCR to detect the *Ripk3* mRNA expression in CD4^+^ T cells, CD11b^+^Ly6G^+^ neutrophils, CD11b^+^Ly6G^-^Ly6C^high^ inflammatory monocytes and CD45^-^CD31^-^ Podoplanin^+^ FLS in inflamed joints (n = 4 each). (**C**) The IL-1β concentration of culture supernatant of BMDCs at 16 h after LPS stimulation (n = 4 each) (**D**) Quantitative RT-PCR to detect the expression of *Ripk3* in the indicated organs of BALB/c mice housed under SPF or GF conditions (n = 4 each). (**E**,**F**) Flow cytometry of IL-17A^+^, GM-CSF^+^, IFN-γ^+^ (n = 3 each) and Foxp3^+^ cells (n = 4 each) in CD4^+^ T cells in Peyer’s patches of WT and *Ripk3*^−/−^ SKG mice. The vertical bars denote the SD in panel **(A–C,F)** and SEM in panel **(D)**. *P < 0.05. Data are pooled from two independent experiments in panels (**A–D,F)**.

Next, we similarly investigated a role of Ripk3 in CD4^+^ T cell homeostasis and the SKG arthritis model. Consistent with the previous report^22^, the expression of *Ripk3* was significantly increased in inflamed joints (Fig. 3A). We further assessed the *Ripk3* expression in CD4^+^ T cells, CD11b^+^Ly6G^+^ neutrophils, CD11b^+^Ly6G^-^Ly6C^high^ inflammatory monocytes and CD45^-^CD31^-^Podoplanin^+^ FLSs sorted from the inflamed joints. *Ripk3* was widely expressed by those key inflammatory cells in SKG arthritic joints, and the expression pattern was different from Gsdmd (Fig. 3B). We then generated *Ripk3*^−/−^ SKG mice on a BALB/c background using a CRISPR/Cas9 system to examine the effects of Ripk3 on the differentiation and maintenance of CD4^+^ T cell subsets in homeostatic and inflammatory conditions (Supplementary Fig. 1B). Like *Gsdmd*^*-/-*^ SKG mice, *Ripk3*^−/−^ SKG mice showed no overt phenotypic changes in development or longevity. We confirmed the loss of the Ripk3 function in necroptosis by culturing BMDCs from *Ripk3*^−/−^ *Rag2*^−/−^ mice with LPS, which showed a significant reduction in the production of IL-1β in comparison to WT *Rag2*^−/−^ mice (Fig. 3C), as previously reported^23^. When the physiological *Ripk3* expression levels in normal tissues from BALB/c mice maintained under SPF or GF conditions were assessed, the *Ripk3* expression was relatively high in the gut, LNs, and spleen (Fig. 3D). Among them, the expression of *Ripk3* in the small intestine was likely controlled by the gut microbiota because of the specific reduction of its expression in GF mice (Fig. 3D). However, we found no difference in the frequency of the IL-17A, GM-CSF, IFN-γ and Foxp3 expression in the CD4^+^ T cells of Peyer’s patches between WT and *Ripk3*^−/−^ SKG mice (Fig. 3E, F). Taken together, these data indicated that Ripk3 did not affect the differentiation or maintenance of physiological CD4^+^ T cell subsets under a homeostatic state.

### Ripk3 is dispensable for the induction and development of SKG arthritis

**Figure 4:**
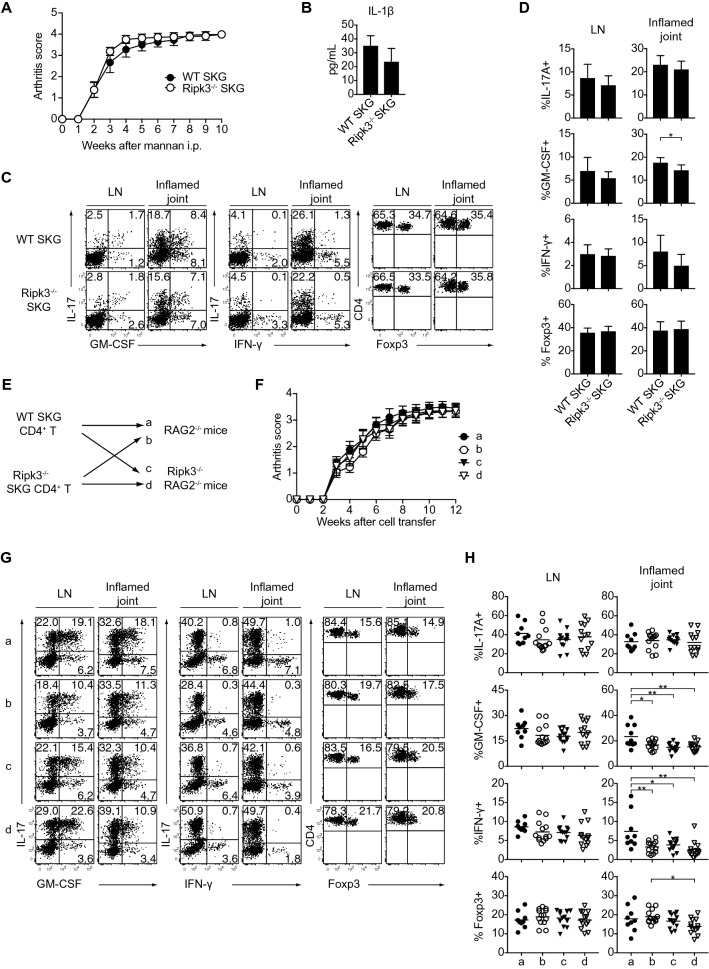
The dispensable role of Ripk3 in the development of autoimmune arthritis*. *(**A**) Arthritis scores of WT (n = 9) and *Ripk3*^−/−^ SKG mice (n = 8) after the injection of mannan (20 mg). (**B**) The IL-1β concentration of synovial tissues from inflamed joints. **(C,D)** Flow cytometry of IL-17A^+^, GM-CSF^+^, IFN-γ^+^ and Foxp3^+^ cells in CD4^+^ T cells in draining LNs and the inflamed joints of WT (n = 9) and *Ripk3*^−/−^ SKG mice (n = 8) at 10 weeks after mannan injection. **(E)** Experimental design of the adoptive transfer of CD4^+^ T cells from WT or *Ripk3*^−/−^SKG mice into *Rag2*^−/−^ or *Ripk3*^−/−^*Rag2*^−/−^ mice. (**F**) The arthritis scores of the four groups (a–d) shown in (**E**) (n = 9 or 13). (**G**,**H**) Flow cytometry of IL-17A^+^, GM-CSF^+^, IFN-γ^+^ and Foxp3^+^ cells in CD4^+^ T cells in draining LNs and inflamed joints of the four groups of mice 12 weeks after cell transfer (n = 9 or 13). The vertical bars denote the SD in panels (**B**,**D**) and SEM in (**A**,**F**). The horizontal line in panel (**H)** denotes the mean. *P < 0.05, **P < 0.01. Data are pooled from two independent experiments in panels (**A**,**B**,**D**,**F**,**H)**.

We next investigated the role of Ripk3 in the induction and development of SKG arthritis. We induced arthritis in WT and *Ripk3*^−/−^ SKG mice. There was no significant difference in the clinical arthritis scores of the two groups of mice (Fig. 4A). Unlike the in vitro assay, IL-1β could be secreted in the inflamed synovium of *Ripk3*^−/−^ SKG mice (Fig. 4B). The frequencies of IFN-γ-, GM-CSF-, IL-17-, and Foxp3-expressing subsets in CD4^+^ T cells in inflamed joints and draining LNs were almost comparable between the groups (Fig. 4C, D). In addition, adoptive transfer experiments using CD4^+^ T cells from WT or *Ripk3*^−/−^SKG mice also revealed no significant differences in the clinical arthritis scores or the proportions of arthritogenic Th17 cells in draining LNs and inflamed joints from the recipient *Rag2*^−/−^ or *Ripk3*^−/−^*Rag2*^−/−^ mice, albeit a marginal decrease in the proportions of IFN-γ- and GM-CSF-producing Th cells in the inflamed joints (Fig. 4E–H). Taken together, these data indicated that Ripk3 is dispensable in the induction of pathogenic Th17 cells and the development of chronic arthritis in SKG mice.

### Crosstalk or the compensatory role between Gsdmd and Ripk3 is not required for the induction and development of SKG arthritis

**Figure 5:**
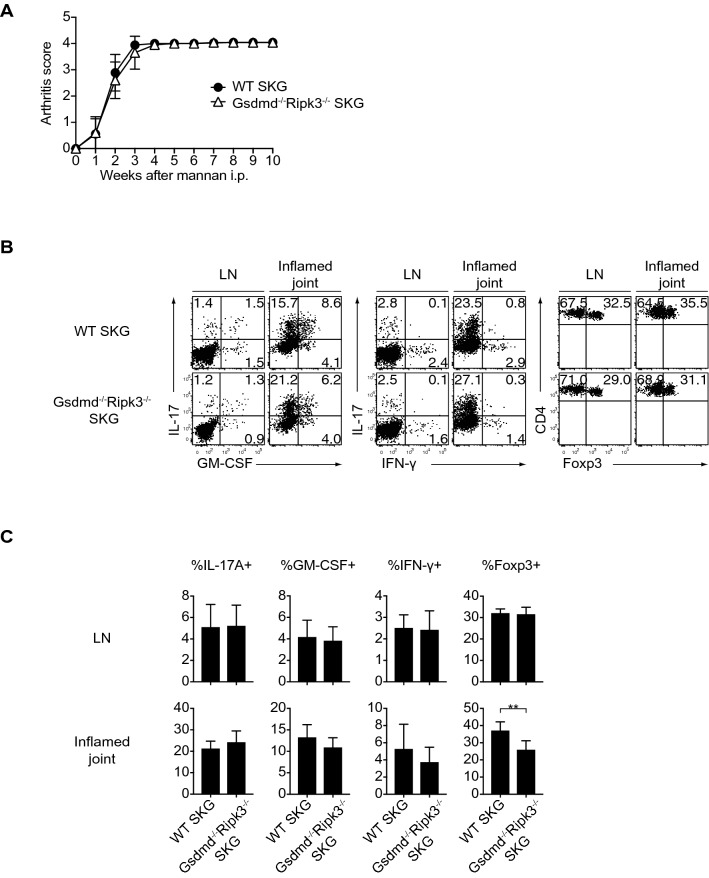
The dispensable role of crosstalk between Gsdmd and Ripk3 in the development of autoimmune arthritis. (**A**) Arthritis scores of WT (n = 9) and *Gsdmd*^−/−^ *Ripk3*^−/−^ SKG mice (n = 10) after the injection of mannan (20 mg). (**B**,**C**) Flow cytometry of IL-17A^+^, GM-CSF^+^, IFN-γ^+^ and Foxp3^+^ cells in CD4^+^ T cells in draining LNs and inflamed joints of WT (n = 9) and *Gsdmd*^−/−^ *Ripk3*^−/−^ SKG mice (n = 10). The vertical bars denote the SD in panel (**C**) and the SEM in (**A**). **P < 0.01. Data are pooled from two independent experiments in panels (**A**,**C)**.

Because a compensatory role and context-dependent interaction between Gsdmd and Ripk3 have been described^24,25^, we generated *Gsdmd*^−/−^ *Ripk3*^−/−^ SKG mice by crossing *Gsdmd*^−/−^ SKG and *Ripk3*^−/−^ SKG mice to assess whether Ripk3 or Gsdmd could compensate for a Gsdmd- or Ripk3-deficient condition, respectively, in the development of autoimmune arthritis, and evaluated their arthritis phenotypes and the development of Th17 cells in *Gsdmd* and *Ripk3* double-deficient conditions. The arthritis scores of *Gsdmd*^−/−^ *Ripk3*^−/−^ SKG mice after the induction of arthritis were unchanged from those in WT SKG mice (Fig. 5A). The differentiation and expansion of IFN-γ-, GM-CSF-, and IL-17-producing Th subsets from the inflamed joints and draining LNs of *Gsdmd*^−/−^ *Ripk3*^−/−^ SKG mice were similar to those of WT SKG mice, albeit with a marginal decrease in the proportions of Foxp3^+^ Treg cells (Fig. 5B, C). Collectively, these results further strengthened the concept that both Gsdmd and Ripk3 and their interactions are dispensable for the induction and development of chronic autoimmune arthritis in SKG mice.

## Discussion

Although accumulated evidence strongly supports that pyroptosis and necroptosis have evolved to combat against infectious pathogens that escape detection by infection-induced apoptosis, particularly intracellular bacteria such as *Shigella flexneri*, the subsequent acute tissue inflammation and pro-inflammatory cytokine production triggered by the programmed cell death could mediate the immunopathology through the aberrant activation of various inflammatory cells^1–4,9–14^. It is noteworthy that the Ripk3 and MLKL—molecules that are specifically involved in necroptosis—were reported to be upregulated in the inflamed synovium of collagen-induced arthritis, indicating a possible contribution of necroptosis to the formation of severe synovitis^22^. How programmed necrosis contributes to T cell-mediated autoimmune arthritis remains poorly understood. Thus, one of the key questions we addressed in the present study was whether the pyroptosis and necroptosis pathways would exacerbate chronic inflammation, in particular sterile synovial inflammation. Using *Gsdmd*^−/−^, *Ripk3*^−/−^, or *Gsdmd*^−/−^ *Ripk3*^−/−^ mice, we demonstrated that despite abundant cell death in inflamed joints, pyroptosis and necroptosis are not required for the expansion of arthritogenic Th17 cells or the progression of Th17-mediated autoimmune arthritis.

Inflammatory mediators, including DAMPs and pro-inflammatory cytokines released by pyroptosis and necroptosis could amplify Th17 functions; however, their modulation of pathogenic Th17 cells seems to be dependent on the type of inflammation and the target organ. Lytic forms of cell death seemed to augment systemic auto-inflammation in an FMF model, and acute tissue injuries in ischemia and transplantation models through the excess production of IL-1β and DAMPs, which could mediate pathology by the activation of innate immune cells^10,12–14^. Given that the pathogenesis of these models is less dependent on adaptive T cell functions, pyroptosis and necroptosis are probably involved in the exacerbation of acute inflammation and tissue injuries are often autonomously amplified by the activation of innate immune and mesenchymal cells. On the other hand, the role of pyroptosis and necroptosis in T cell-mediated autoimmune pathology may be dispensable or limited in a specific inflammatory context. EAE is an exceptional model which relies on Gsdmd for the development of Th17-mediated autoimmune neuroinflammation. Mechanistically, Gsdmd-dependent pyroptosis of the peripheral myeloid cells is necessary for the activation and differentiation of pathogenic Th cells in peripheral lymphoid tissues, which in turn migrate into the target spinal cord to cause chronic tissue inflammation^11^. Although pathogenic Th17 cells predominantly drive SKG arthritis and EAE, the differential requirement of Gsdmd in the models may be attributed how these diseases are induced. For EAE, immunization with complete Freund’s adjuvant and pertussis toxin, potent bacterial compounds, may be a strong inducer of pyroptosis in myeloid cells, following the activation of inflammasomes. In contrast, SKG mice are a spontaneous arthritis model and can be induced by the injection of mannan, which is a fungal compound distinct from that used in EAE and may not be involved in the activation of the pyroptosis pathway. Therefore, T cell priming in peripheral LNs between the models is probably different and Gsdmd was not required for the induction of arthritogenic Th17 cells in the LNs. However, it is tempting to speculate that persistent IL-1β production and Th17-mediated chronic inflammation in the target tissues of EAE or SKG mice may be similarly independent of pyroptosis and necroptosis, despite abundant cell death occurring in the inflamed tissue.

Crosstalk among different forms of programmed cell death, including apoptosis and necrosis, has recently been highlighted as a new aspect of interactions and compensatory roles between the signaling pathways of the distinct programs^24–26^. Although there are various triggers (TNF, endogenous inflammasome ligands, etc.) of pyroptosis and necroptosis in inflamed joints, our data using *Gsdmd*^−/−^ *Ripk3*^−/−^ SKG mice clearly excluded the compensatory role between Gsdmd and Ripk3 for the development of autoimmune arthritis and further strengthened our claim that they are not involved in Th17 cell-mediated chronic inflammation. However, considering the IL-1β production capability in *Gsdmd*^−/−^ and *Ripk3*^−/−^ mice and its crucial role in SKG arthritis^27^, unappreciated cellular mechanisms that can also process IL-1β maturation and release may be present in sterile tissue inflammation (e.g., the synovium and central nervous system)^28,29^.

In contrast, given that *Gsdmd*^−/−^ and *Ripk3*^−/−^ mice do not show an overt phenotype in development or physiology, inhibitors of Gsdmd or Ripk3 may be a potential therapeutic target for the treatment of innate immune cell-mediated tissue injuries and systemic auto-inflammation because the inhibitors could have limited adverse effects with regard to the blocking of physiological functions and T cell-mediated immunity. Further studies are needed to investigate the unknown mechanisms through which programmed cell death modalities are involved in the production of pro-inflammatory mediators (*e.g*., IL-1β), and the amplification of chronic tissue inflammation in autoimmune diseases.

## Materials and methods

### Mice

SKG and *Rag2*^−/−^ mice were described previously^21^. *Gsdmd*^−/−^ and *Ripk3*^−/−^ SKG mice were generated using a CRISPR/Cas9 system. *Gsdmd*^−/−^ *Rag2*^−/−^, *Ripk3*^−/−^ *Rag2*^−/−^, and *Gsdmd*^−/−^ *Ripk3*^−/−^ SKG were generated by crossing the above described strains. BALB/c mice housed under SPF and GF conditions were purchased from CLEA Japan. All animal experiments were approved by the Ethical Committee of Institute for Frontier Life and Medical Sciences and Graduate School of Medicine, Kyoto University, and were performed in compliance with the institutional and ARRIVE guidelines.

### Induction of arthritis

Arthritis was induced by a single intraperitoneal injection of mannan (20 mg; Sigma-Aldrich) in SKG mice, or by adoptive transfer of SKG CD4^+^ T cells (intravenously) into recipient mice, and was scored as described previously^19,20,30^. For the adoptive transfer of CD4^+^ T cells, CD4^+^ T cells were positively selected from the spleen and peripheral LNs using MACS CD4 microbeads and an LS column (Miltenyi Biotec) according to the manufacturer’s instruction, and 2 × 10^6^ CD4^+^ T cells were transferred into each recipient mouse intravenously via the tail vein.

### Single cell suspension from synovial tissue and flow cytometry

The collected synovial tissues were cut into small pieces, followed by enzymatic digestion for 1 h at 37 °C in 3 ml of IMDM buffer containing 2% FBS (GIBCO) with 300U of collagenase I and collagenase IV (Worthington). Digested tissues were then mashed through a 70-μm mesh filter. The resultant single cell suspension of the synovial tissues was used for flow cytometry and cell sorting experiments. For intracellular staining of cytokines, cells were restimulated for 2.5 h with phorbol 12-myristate 13-acetate (50 ng/ml; Sigma-Aldrich) and ionomycin (500 ng/ml; Sigma-Aldrich) in the presence of Brefeldin A (1 μg/ml; Merck) in IMDM (Sigma-Aldrich) supplemented with 5% FBS (GIBCO), 2-mercaptoethanol (GIBCO), GlutaMAX (GIBCO), sodium pyruvate (GIBCO), MEM NEAA (GIBCO) and penicillin–streptomycin (Nacalai Tesque). Cells were then fixed with 3.7% formaldehyde (Sigma-Aldrich), followed by permeabilization with 0.1% NP-40 (Nacalai Tesque), and staining with antibodies against cytokines. Foxp3 was stained using the Foxp3/Transcription Factor Staining Buffer Set (eBioscience) in accordance with the manufacturer’s instructions. The following monoclonal antibodies were used for flow cytometry (BD FACSCantoII) and cell sorting (SONY MA900): anti-mouse CD4 (RM4-4), CD11b (M1/70), CD45.2 (104), Ly-6G (1A8), Podoplanin (8.1.1), IL-17A (TC11-18H10.1), GM-CSF (MP1-22E9), and IFN-γ (XMG1.2), all from Biolegend, anti-mouse Ly-6C (AL-21), CD31 (390) and PE-Streptavidin, all from BD Bioscience, anti-mouse Foxp3 (FJK-16 s) from eBioscience. All FACS data were analyzed using the FlowJo software program (Tree Star, Inc.).

### Quantitative RT-PCR

Total RNA from tissues and sorted cells was extracted using TRIzol (InvitroGen) and was reverse-transcribed with SuperScript VILO (Invitrogen). The resultant cDNA was used for quantitative RT-PCR on a Light cycler 480 (Roche) or StepOnePlus (Applied Biosystems) using qPCR master mix (TOYOBO) or Luna Universal qPCR Master Mix (NEB) and TaqMan Gene Expression Assays (Thermo Fisher: *Gsdmd,* Mm00509958_m1; *Ripk3* Mm00444947_m1; *Hprt*, Mm03024075_m1). The expression levels of target genes were quantified after normalization to the expression of *Hprt* as a housekeeping gene.

### Generation of bone marrow-derived dendritic cells (BMDCs)

Bone marrow cells were collected from the femur and tibia and red blood cells were lysed with RBC lysis buffer (Sigma-Aldrich). Resultant cells were cultured at 5 × 10^5^/ml in RPMI containing 10% FBS, 2-mercaptoethanol and penicillin–streptomycin with 20 ng/ml GM-CSF for 6 days to differentiate into BMDCs for in vitro assays.

### Measurement of IL-1β from BMDCs and joint tissues

BMDCs were cultured in 96-well plates at 1 × 10^5^ cells per well and either treated with 1 μg/ml LPS (InvivoGen), or transfected with 1 μg/ml LPS using 0.3% v/v FuGENE HD (Promega) after priming with 1 μg/ml Pam3CSK4 (InvivoGen) for 6 h. At 16 h after LPS stimulation, the IL-1β concentrations in the culture supernatants were measured using a Cytometric Bead Array (BD Biosciences) in accordance with the manufacturer’s instructions. As for the measurement of IL-1β in the synovium, synovial tissues were resected from the inflamed joints, cut into small pieces, and the tissue weight was measured. The resultant synovial tissues were placed into 500 μl of D-PBS (−) (Nacalai Tesque), then vortexed and centrifuged. The collected supernatants were then used for the measurement of the IL-1β concentration, which was performed using a Cytometric Bead Array, and the results were normalized with tissue weights of individual samples.

### Statistical analysis

Statistical analyses were performed using the GraphPad PRISM7 software program (GraphPad Software). A two-tailed t-test was used for the statistical analysis. A two-way ANOVA followed by Tukey’s multiple comparisons test was used for the analysis of grouped data. Data are shown as the mean ± standard error of mean (SEM) or mean ± standard deviation (SD). *P* values of < 0.05 were considered to indicate statistical significance.

## Supplementary Information

Supplementary Figure S1.

## Data Availability

The datasets analyzed during the current study are available from the corresponding author on reasonable request.
