# Supplementary material for: Dispensable roles of Gsdmd and Ripk3 in sustaining IL-1β production and chronic inflammation in Th17-mediated autoimmune arthritis
Source: Sci Rep. 2021 Sep 21;11:18679. doi: 10.1038/s41598-021-98145-y (PMC8455622; doi:10.1038/s41598-021-98145-y)
Supplement: Supplementary file 1 — Supplementary Figure S1. [file 41598_2021_98145_MOESM1_ESM.pdf]

Supplementary Information

Title:Dispensable roles of Gsdmd and Ripk3 in sustaining IL-1β production and chronic inflammation in Th17-mediated autoimmune arthritis

Authors: Yusuke Takeuchi, Daiya Ohara, Hitomi Watanabe, Noriko Sakaguchi, Shimon Sakaguchi, Gen Kondoh, Akio Morinobu, Tsuneyo Mimori, Keiji Hirota

Supplementary Figure 1

A

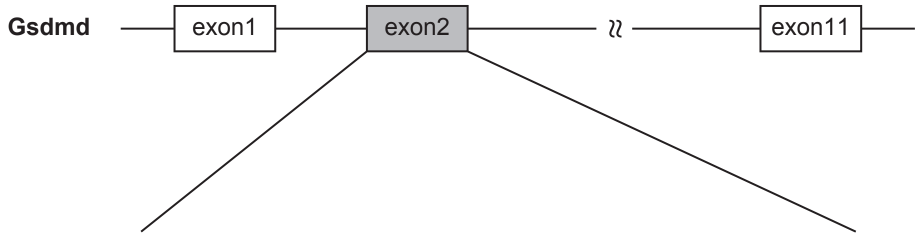

ATGCCATCGGCCTTTGAGAAAGTGGTCAAGAATGTGATCAAGGA
GGTAAGCGGCAGCAGAGGCGATCTCATTCCGGTGGACAGCCTG
CGGAACTCCACCAGCTTCAGGCCCTACTGCCTTCTGAACAGGAA
ATTTTCAAGCTCAAGGTTCTGGAAACCCCGTTATTCATGTGTCAA
CCTGTCAATCAAGGACATCCTGGAGCCCAGTGCTCCAGAACCAG

B

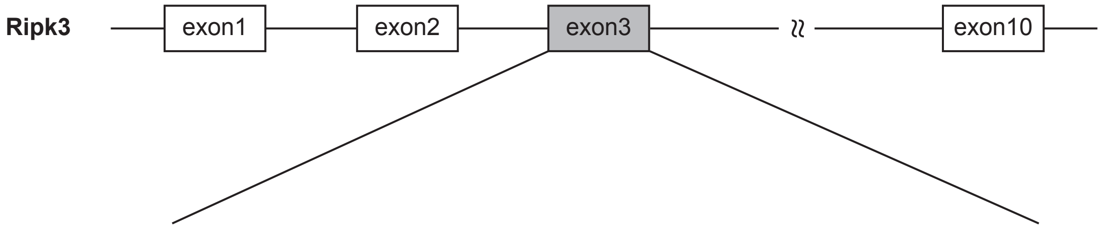

GAAGAAGATATCCTGGGAGGTGAAGGCTATGGTTAATCTTCGTAATGAGAACG
TTCTGCTCCTGCTGGGGGTCACTGAGGACCTCCAGTGGGACTTCGTGTCCGG
GCAGGCTCTGGTGACAAGATTCATGGAGAATGGCTCCCTCGCAGGGCTGCTG
CAACCCGAGTGCCCTCGGCCCTGGCCACTCCTCTGTCGCCTGCTGCAGGAAG
TGGTGCTGGGGATGTGCTACCTACACAGCTTGAACCCTCCGCTCCTGCACCG
GGACCTCAAGCCCTCTAACATTCTGCTGGATCCAGAGCTCCACGCCAAG

Supplementary Fig.1. Genome editing with CRISPR/Cas9 system to generate *Gsdmd*$^{-/-}$ and *Ripk3*$^{-/-}$ mice.
(A, B) Frameshift mutations were made with CRISPR/Cas9 system by deleting 22bp (CGGTGGACAGCCTGCGGAACTC) in exon 2 of Gsdmd gene and 28bp (CAGGGCTGCTGCAACCCGAGTGCCCTCG) in exon 3 of Ripk3 gene to generate *Gsdmd*$^{-/-}$ and *Ripk3*$^{-/-}$ mice, respectively.
